# An integrin αEβ7-dependent mechanism of IgA transcytosis requires direct plasma cell contact with intestinal epithelium

**DOI:** 10.1038/s41385-021-00439-x

**Published:** 2021-08-20

**Authors:** Mauricio Guzman, Luke R. Lundborg, Shaila Yeasmin, Christopher J. Tyler, Nadia R. Zgajnar, Vanessa Taupin, Katarzyna Dobaczewska, Zbigniew Mikulski, Giorgos Bamias, Jesús Rivera-Nieves

**Affiliations:** 1 Gastroenterology Section, San Diego VA Medical Center, La Jolla Village Drive, San Diego, CA USA; 2 Division of Gastroenterology, Department of Medicine, University of California San Diego, La Jolla, CA USA; 3 Electron Microscopy Core Facility, Department of Cellular and Molecular Medicine, University of California San Diego, La Jolla, CA USA; 4 Microscopy and Histology Core, La Jolla Institute of Allergy and Immunology, La Jolla, CA USA; 5 GI Unit, 3rd Academic Department of Internal Medicine, National and Kapodistrian University of Athens, Sotiria Hospital, Athens, Greece

## Abstract

Efficient IgA transcytosis is critical for the maintenance of a homeostatic microbiota. In the canonical model, locally-secreted dimeric (d)IgA reaches the polymeric immunoglobulin receptor (pIgR) on intestinal epithelium via simple diffusion. A role for integrin αE(CD103)β7 during transcytosis has not been described, nor its expression by intestinal B cell lineage cells. We found that αE-deficient (αE^−/−^) mice have a luminal IgA deficit, despite normal antibody-secreting cells (ASC) recruitment, local IgA production and increased pIgR expression. This deficit was not due to dendritic cell (DC)-derived retinoic acid (RA) nor class-switching defects, as stool from RAG^−/−^ mice reconstituted with αE^−/−^ B cells was also IgA deficient. Flow cytometric, ultrastructural and transcriptional profiling showed that αEβ7-expressing ASC represent an undescribed subset of terminally-differentiated intestinal plasma cells (PC) that establishes direct cell to cell contact with intestinal epithelium. We propose that IgA not only reaches pIgR through diffusion, but that αEβ7+ PC dock with E-cadherin-expressing intestinal epithelium to directly relay IgA for transcytosis into the intestinal lumen.

## Introduction

Secretory Immunoglobulin A (SIgA) is critical for the control of the intestinal microbiota. Therefore, luminal IgA levels are tightly maintained by a sequence of processes that include B cell progenitor production at the bone marrow, naïve B cell migration to inductive sites, (e.g., Peyer Patches (PP)), immunoglobulin (Ig)A class-switching in germinal centers, IgA plasmablast egress and recruitment into intestinal lamina propria (LP), maturation/survival of plasma cells (PC), local IgA production by antibody-secreting cells (ASC), and polymeric Immunoglobulin receptor (pIgR)-mediated epithelial transcytosis into the intestinal lumen. Both integrin β7- and MAdCAM-1-deficient mice exhibit underdeveloped PP and an IgA + ASC deficit in the LP, underlining the importance of integrin α4β7:MAdCAM-1 interactions for naïve B cell migration to PP and intestinal recruitment of IgA plasmablasts.^1–3^ In contrast, the expression and functions of integrin αE(CD103)β7 appear to be much narrower.

αEβ7 was first described as the HML-1 antigen generated by immunization of mice with human intestinal intraepithelial lymphocytes (IEL).^4^ Subsequently, the antibodies HML-1 and B-Ly7 were identified to recognize the same molecule: integrin αE,^5^ expressed abundantly on the cell surface of malignant B cells. CD103 is widely used as a surface marker for hairy cell leukemia, a B cell cancer.^6^ αEβ7 is expressed by intraepithelial lymphocytes (IEL)^7^ and mediates their interactions with intestinal epithelial cells (IEC) via E-cadherin.^8,9^ A mucosal dendritic cell (DC) subset also expresses αEβ7.^10^ This DC subset was later found to be a major producer of retinoic acid (RA)^11^, critical for induction of a gut-homing phenotype, regulatory T cells (Treg) and IgA class-switching.^12–14^ However, the physiologic role of the integrin in this DC subset remains unclear, as CD103^−/−^ DC are not impaired on their ability to imprint a gut-homing phenotype to T cells.^15^ αEβ7 has also been reported in a subset of B cells at the nasal mucosa and the head and neck.^16,17^ In the intestine, by contrast, neither its expression by cells of the B cell lineage nor its potential involvement in IgA luminal transport have been recognized.^18^ Here, we report on an undescribed subset of terminally-differentiated αEβ7-expressing IgA^+^ PC that establish direct contact with E-cadherin/pIgR-expressing IEC. We identify a new role for αEβ7 during IgA transcytosis and propose a novel mechanism of direct IgA relay to IEC by PC for its transcytosis into the intestinal lumen.

## Results

### Fecal IgA is lower in integrin αE-deficient mice despite normal B cell recruitment, IgA production and increased pIgR mRNA expression

**Fig. 1:**
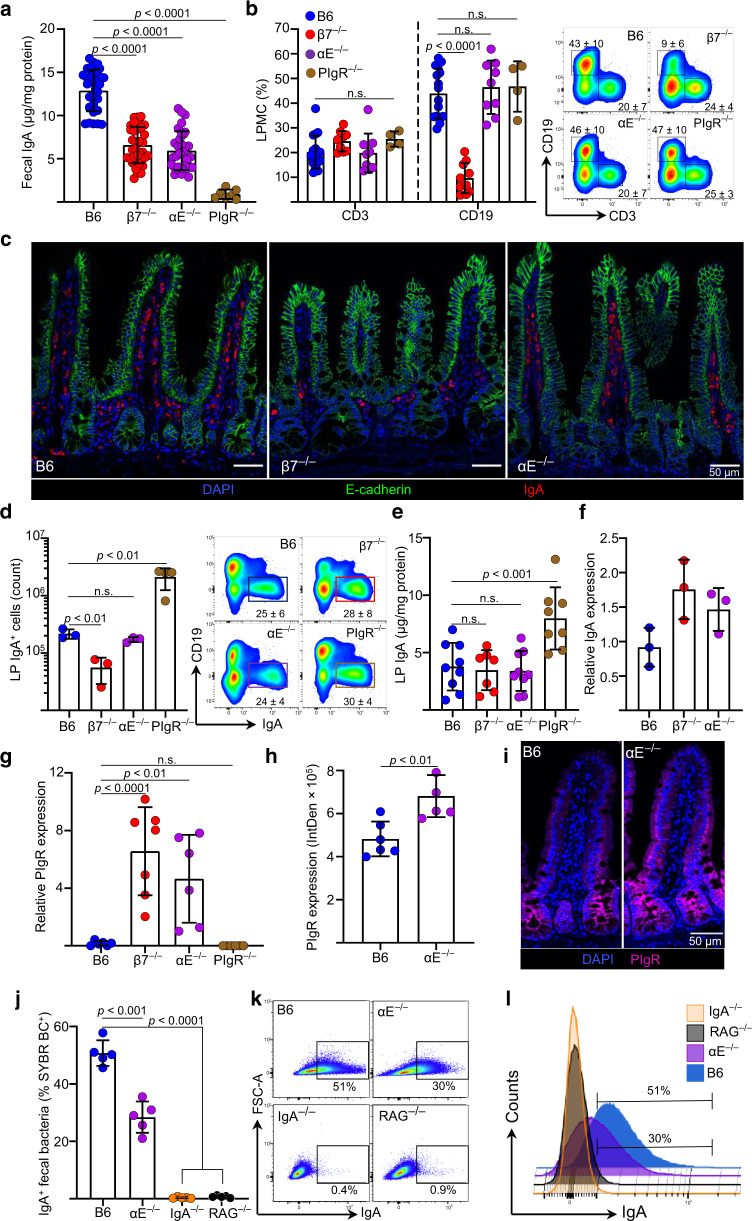
Integrin αE deficiency leads to a fecal IgA deficit despite normal B cell recruitment, IgA production and increased pIgR mRNA expression. **a** Fecal IgA levels measured by ELISA in C57BL/6 (WT), Itgb7^−/−^ (β7^−/−^), Itgae^−/−^ (αE^−/−^) and pIgR-deficient (pIgR^−/−^) mice. **b** Percentage of CD3+ and CD19+ cells within the ileal LP of indicated strains and representative contour plots of indicated cell subsets gated on live, single cellular events. **c** IF staining of IgA + ASC and E-cadherin, representative coronal images of terminal ileum (TI). **d** Absolute numbers and representative dot plots of IgA + ASCs in lamina propria. **e** LP IgA levels measured by ELISA of indicated strains (**f**) ileal IgA mRNA transcripts in ilea of indicated mice (**g**) pIgR mRNA expression in ileum of indicated mice. **h** Relative pIgR fluorescence intensity expressed as integrated density (IntDent, see “Methods”) per villi and (**i**) representative IF images (**j**) Percentage of IgA-coated fecal bacteria measured by flow cytometry and (**j**, **k**, **l**) representative plots and histograms of IgA coating of indicated mouse strains. Each data point (mean ± SD) represent a single mouse with *n* ≥ 5 from 2 or 3 independent experiments. Statistical significance determined using two-way ANOVA with Dunnett’s multiple comparisons test.

β7-deficient mice (β7^−/−^) have dual integrins α4β7 and αEβ7 deficits. IgA ASC are decreased in their intestinal lamina propria. This finding is attributable to the absence of α4β7/MAdCAM-1-mediated ASC recruitment rather than to the αEβ7 defect, as this is also observed in MAdCAM-1-deficient mice^1,3^. The maintenance of luminal secretory (S)IgA levels is dependent on several processes that include: 1. IgA plasmablast recruitment, 2. local IgA production and 3. pIgR-mediated transcytosis. We measured fecal SIgA as a surrogate indicator of the integrity of these processes in C57BL6 (B6), β7^−/−^ and αE^−/−^ mice, using pIgR-deficient mice (pIgR^−/−^) as controls. Unexpectedly, not only β7^−/−^ but also αE^−/−^ mice had lower fecal IgA than B6 mice (Fig. 1a). To exclude a recruitment deficit, we analyzed the mononuclear cell composition of their ileal LP. CD19^+^ B cells were decreased only in the LP of β7^−/−^, but not in αE^−/−^ mice. By contrast, the percentage of CD3+ T cells was uniformly unaffected (Fig. 1b), suggesting alternate integrin use by T cells for intestinal recruitment. Immunofluorescence (IF) confirmed impaired IgA^+^ ASC recruitment only in β7^−/−^ mice but not in αE^−/−^ mice (Fig. 1c). The T and B cell composition of the spleen and mesenteric lymph nodes (MLN) was not different between any strain, in support of an intestinal-specific B cell recruitment deficit in β7^−/−^ mice (Supplementary Fig. 1a, b). Absolute counts of IgA^+^, IgD^+^ and IgM^+^ ASC were also lower only in β7^−/−^ mice, as shown previously for IgA^+^ ASC^1^ (Fig. 1d, Supplementary Fig. 1c, d). Thus, all ASC were reduced in LP of β7^−/−^ mice, not only IgA^+^ ASC.

We then evaluated whether defective local IgA production could account for the luminal IgA deficit. IgA levels within the intestinal LP was similar between B6, β7^−/−^ and αE^−/−^ mice (Fig. 1e), whereas pIgR^−/−^ mice had higher levels, as reported previously.^19^ CD19^+^ B cells from all strains also showed equal ability to IgA class-switch ex vivo (Supplementary Fig. 1e, f). Given the remarkable ability of β7^−/−^ mice to maintain luminal SIgA to a level comparable to that of αE^−/−^ mice, despite their marked recruitment deficit, we examined their IgA mRNA transcripts. These were higher in β7^−/−^ mice, suggesting that IgA + ASC may sense and adapt to the luminal IgA deficit by enhancing their production of IgA (Fig. 1f).

As all intraluminal IgA is produced locally and transcytosed by pIgR, we excluded a pIgR deficit as an explanation for the low luminal IgA by quantifying ileal pIgR mRNA transcripts.^20^ Transcripts were actually higher in both β7^−/−^ and αE^−/−^ mice, compared with B6 and pIgR^−/−^ mice (Fig. 1g). pIgR upregulation is a second mechanism through which these strains maintain luminal IgA levels. We confirmed PIgR protein overexpression by IF (Fig. 1h, i). Increased fluorescent signal was quantified in an unbiased fashion using Fiji ImageJ software. The product of the area and mean intensity (Int Den) were recorded for B6 and αE^−/−^ mice (Fig. 1h).

Finally, to begin to understand possible in vivo implications of a potential αE-dependent transcytosis defect, we compared fecal bacterial IgA coating in αE^−/−^ and B6 mice, using bacteria from IgA^−/−^ and RAG^−/−^ mice as controls. We observed a significant decrease in bacterial IgA coating in stool of αE^−/−^ mice (Fig. 1j, k, i). Taken together, these findings demonstrate that the fecal IgA deficit in αE^−/−^ mice could not be explained by impaired B cell/IgA ASC recruitment, local IgA production, IgA class switching nor pIgR expression. Instead, integrin αEβ7 appeared to play an unappreciated role for the maintenance of luminal SIgA levels and bacterial IgA coating.

### Transfer of αE-deficient B cells to RAG^−/−^ mice recapitulated the luminal IgA deficit, excluding an αEβ7^+^ dendritic cell-derived retinoic acid deficit as a cause

**Fig. 2:**
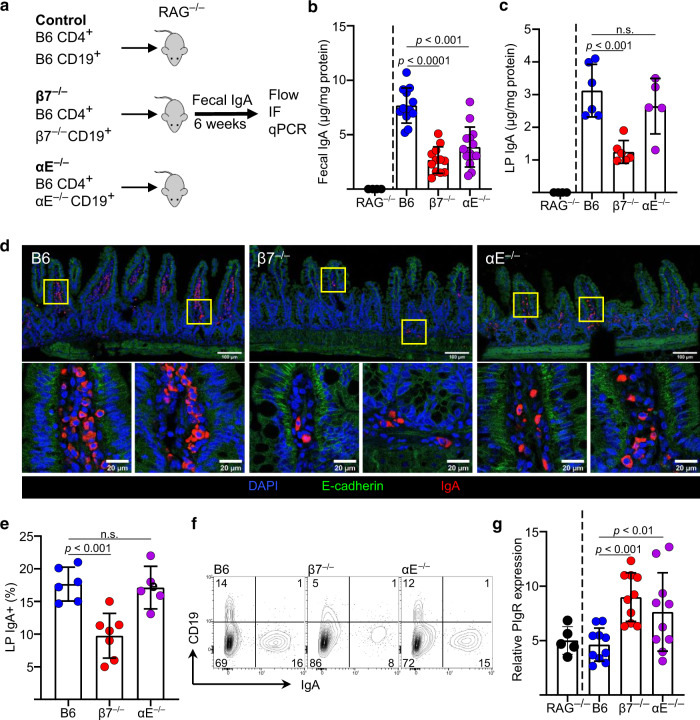
αE-deficient B cells are unable to reconstitute luminal IgA despite normal recruitment, local Ig production and increased PIgR expression. **a** Experimental design of adoptive transfers. **b** Fecal IgA levels measured by ELISA. **c** LP IgA levels of indicated strains measured by ELISA. **d** IF staining of IgA + ASC and E-cadherin (representative coronal TI images) of indicated strains. **e** Percentage of IgA+ cells within the ilea of indicated mouse strains and (**f**) representative contours plots. **g** Ileal pIgR mRNA expression of RAG^−/−^ mice transferred with B cells of indicated strains (mean ± SD, *n* = 6 to 12 mice from 3 independent experiments, significance determined using ANOVA, followed by Tukey’s multiple comparison test.

In 1993 a subset of intestinal DC were found to express integrin αEβ7.^10^ These were later shown to produce RA^11^, an important factor for imprinting a gut-homing (CCR9, α4β7) and regulatory T cell (Treg) phenotypes on lymphocytes and for IgA class-switching in ASC.^12^ To examine whether the luminal IgA deficit in αE^−/−^ and β7^−/−^ mice could be related to the absence of CD103^+^ DC and impaired RA synthesis, we co-transferred B6 CD4^+^ T cells along with CD19^+^ B cells from B6 (controls), β7^−/−^ or αE^−/−^ B cells into RAG^−/−^ mice, which have an intact DC/RA system^21,22^ (Fig. 2a). As observed in donor mice, fecal SIgA was lower in recipients of β7^−/−^ and αE^−/−^ B cells (Fig. 2b). ELISA, flow cytometry and confocal microscopy analysis showed that LP IgA and IgA + ASC were lower only in mice receiving β7^−/−^ B cells, reflective a B cell recruitment deficit only in the latter strain (Fig. 2c–f)). As observed with β7^−/−^ or αE^−/−^ mice, pIgR expression was higher in ilea of mice transferred with either β7^−/−^ or αE^−/−^ B cells (both with luminal IgA deficits) (Fig. 2g), but not in those receiving B6 B cells. The percentage of B and T cells in the spleen and MLN of RAG^−/−^ mice reconstituted with either β7^−/−^ or αE^−/−^ donor cells was normal, as were the percentages of CD4+ T cells in both the LP and periphery (Supplementary Fig. 2a–c), confirming effective T and B cell reconstitution. Thus, our results show that β7^−/−^ B cells, but not αE^−/−^ B cells were impaired in their ability to migrate, populate the LP or IgA class-switch. Yet, the luminal SIgA deficit persisted. Furthermore, by utilizing an experimental system in which only B cells lack the integrin, we exclude a shortfall of RA-producing CD103^+^ DC as the cause for the lower fecal SIgA in β7^−/−^ and αE^−/−^ mice.

### A subset of IgA + ASC expressed αEβ7 and had ultrastructural and transcriptional profiles consistent with that of terminally-differentiated plasma cells

**Fig. 3:**
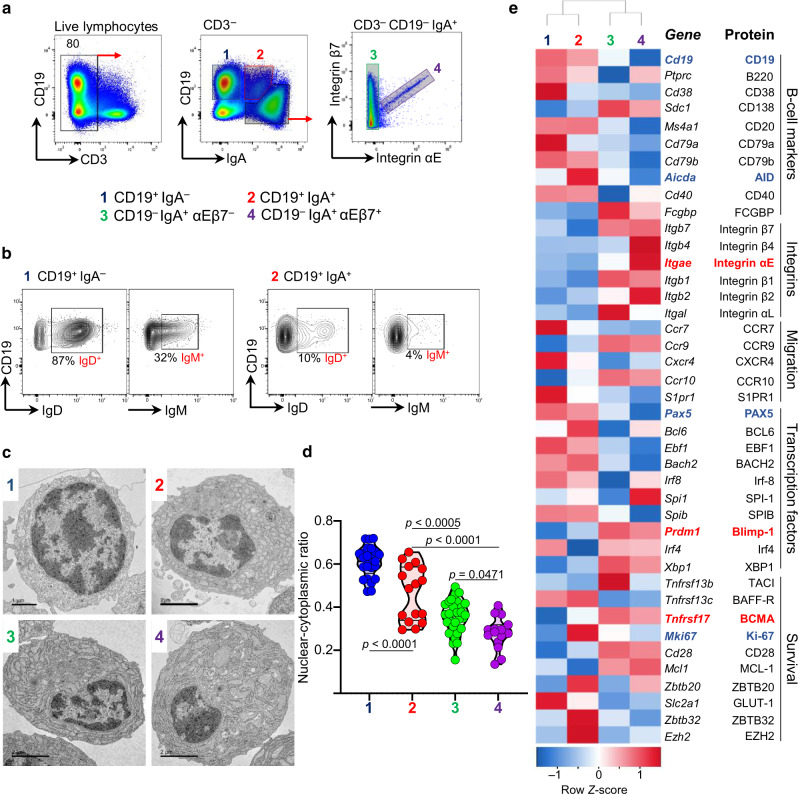
αEβ7+ B cells are a subset of terminally-differentiated PC in small intestinal lamina propria. **a** Sorting strategy for intestinal LP B cell lineage cells. **b** Representative contours plots of IgD and IgM expression in subsets 1 and 2. **c** Distinct ultrastructure of subsets (representative images). **d** Nucleo-cytoplasmic ratio of subsets (violin plot of median and quartiles, *n* > 5, from 3 independent experiments, *p* calculated by one-way ANOVA with Sidak’s correction). **e** Heat map of indicated PC-related genes by RNA-seq (2-way ANOVA, up- or down-regulated >2-fold from 3 independent cell sorts).

Surface αEβ7 protein expression on a subset of CD19^neg^ IgA + ASC was confirmed by flow cytometry in C57BL6 mice, which additionally allowed sorting of ileal LP B cell lineage cells, based on CD19, surface IgA expression and co-expression of integrin subunits αE and β7. We identified 4 main subsets: subset(s) 1 [CD19 ^+ ^IgA^neg^], s2 [class switched CD19^+^ IgA^+^ plasmablasts], s3 [CD19^neg^IgA^+^ αEβ7^neg^ PC], and s4 [CD19^neg^IgA^+^ αEβ7^pos^ PC] (Fig. 3a). S1 was mostly IgD + and a third also IgM positive, suggesting that they were in earlier stages of differentiation (Fig. 3b) and likely originating from inducible lymphoid follicles (ILF). Ultrastructurally, transmission electron microscopy (TEM) showed that their nucleocytoplasmic ratio decreased progressively from s1 through s4(αEβ7^+^). The latter had abundant rough endoplasmic reticulum (RER) and low nucleo:cytoplasmic ratio, consistent with terminal differentiation (Fig. 3c, d).

mRNA was extracted from cell subsets sorted as in Fig. 3a and their transcriptomic profiles analyzed by RNA-seq (Fig. 3e). The expression of B cell-related genes representative of distinct stages of B cell maturation^23^ is shown. Overall, gene expression changed the most upon IgA class switching (CD19^+^ (s1, 2) vs. CD19^neg^IgA + (s3, 4)). IgA class-switched (s3,4) lacked CD19, known to be shed upon class switching. Similarly, they lacked other surface proteins that are lost with maturation, such as CD38 (lost in mouse PC, not human) and CD20. The CD79a and CD79b proteins form a dimer associated with membrane-bound immunoglobulin, constituting the B cell receptor (BCR), which disappears in the later stages of PC differentiation. Along those lines the costimulatory molecule CD40 on B cells interacts with the CD40L during antigen presentation. The absence of CD40 supports that these cells no longer present antigen, as expected for professional PC. Consistent with a PC identity they expressed syndecan-1 (CD138), a classic marker of mature ASC that binds to extracellular matrix components, integrins, pro-survival cytokines and chemokines. The B220 gene (*Ptprc*) which like CD19 is often shed by most mature PC was present at the mRNA but not at the protein level by flow cytometry (not shown). Interestingly both IgA^+^ subsets possess transcripts for IgGFC binding protein (FCGBP), a characteristic secretory product of most mucin-producing cells including Goblet cells. The molecular function of FCGBP has not been elucidated in detail, but it is likely to regulate pathogen attachment and the clearing of microorganisms. Predictably, both s3 and 4 no longer possess transcripts for activation-induced cytidine deaminase (AID, *Aicda*), an enzyme that mediates somatic hypermutation and class-switch recombination. AID is strongly expressed in s2 (CD19+ IgA+) which are actively class-switching.

αEβ7^+^ PC (s4) additionally possess transcripts for integrin β4 which would allow these cells to bind to laminin. They also expressed gut homing chemokine receptors: CCR9 and CCR10, suggesting they are intestinal-specific. These receptors are involved in epithelial interactions and both of their ligands (CCL25, CCL28) are produced by ileal and colonic epithelial cells. Conversely, they lack S1PR1 and CCR7 (expressed by immature B cells), suggesting that they are tissue residents and do not travel to blood or lymphoid tissues, where their respective ligands S1P1 and CCL19/21 are abundant.

Certain transcription factors expressed by αEβ7^+^ PC include Regulator of plasma cell differentiation *Prdm1* (Blimp1)^24,25^ and *Irf4*, essential for the generation of PC. Their function in mature PC remains poorly understood. X-box binding protein 1 (Xbp1), also expressed by IgA + ASC regulates PC differentiation, whereas Zbtb20 promotes PC differentiation and longevity.^26^ Subsets 1 and 2 expressed *Pax5* and *Bcl6*; genes expressed by germinal center B cells^27^, suggesting that they may originate from inducible lymphoid follicles within the intestine.^28^

Several molecules that promote long-term survival are also expressed by both IgA^+^ ASC (s3,4). These include CD28 (receptor for CD80/86) and myeloid leukemia cell differentiation protein (MCL-1), both enhance survival through the inhibition of apopotosis. *Tnfrsf17*, which encodes for B cell maturation antigen (BCMA is expressed by terminally-differentiated long-lived PC^29^ and recognizes B-cell activating factor (BAFF), which also promotes survival. The absence of ki67 (*MKi67*) demonstrate that they are no longer replicating.

### IgA ASC align with, contact and/or interdigitate within E-cadherin/pIgR-expressing ileal epithelium

**Fig. 4:**
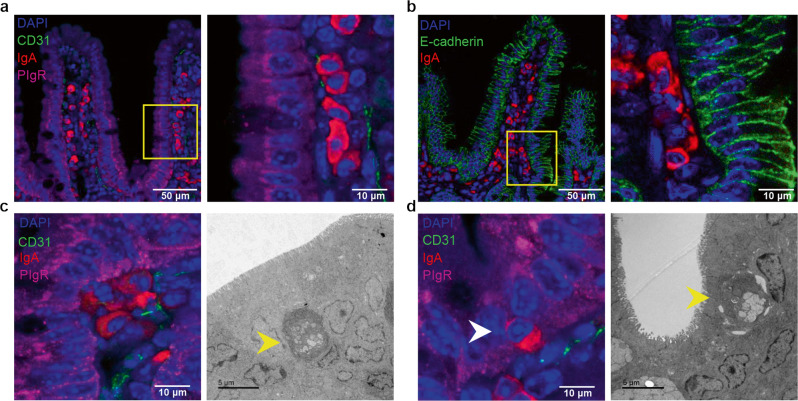
IgA ASCs align, contact and/or interdigitate E-cadherin/pIgR-expressing ileal epithelium. **a** IF staining of IgA + ASC adjacent to pIgR-expressing epithelium (representative images). **b** IgA + ASC adjacent to E-cadherin-expressing epithelium. **c, d** IF and TEM of IgA + ASC in contact and intercalated within pIgR-expressing terminal ileal epithelium (representative images).

Integrin αEβ7 mediates IEL interactions with IEC^8,30^ via E-cadherin, an adhesion molecule expressed on the epithelial basolateral surface.^31^ Recent intravital microscopy studies have shown that IEL are not anchored, but in constant movement, interacting with multiple IEC.^32^ Cells of the B cell lineage are not considered to express αEβ7, although some scarce exceptions have been reported.^4,33^ However, we identify a population of IgA^+^ ASC that align themselves in direct contact with pIgR- and E-cadherin-expressing IEC (Fig. 4a, b). Such IgA ASC rarely localized near the villus tips, where expression of pIgR is low or absent (Fig. 4a), but rather near the crypt base, where they are frequently found in direct contact with IEC (Fig. 4c). In addition, we find certain IgA + ASC that like IEL, intercalate between IEC (Fig. 4d, Supplementary 3a–c) These intraepithelial IgA ASC are absent in αE^−/−^ mice, suggesting that the integrin is required for such intraepithelial positioning (Supplementary Fig. 3c).

**Fig. 5:**
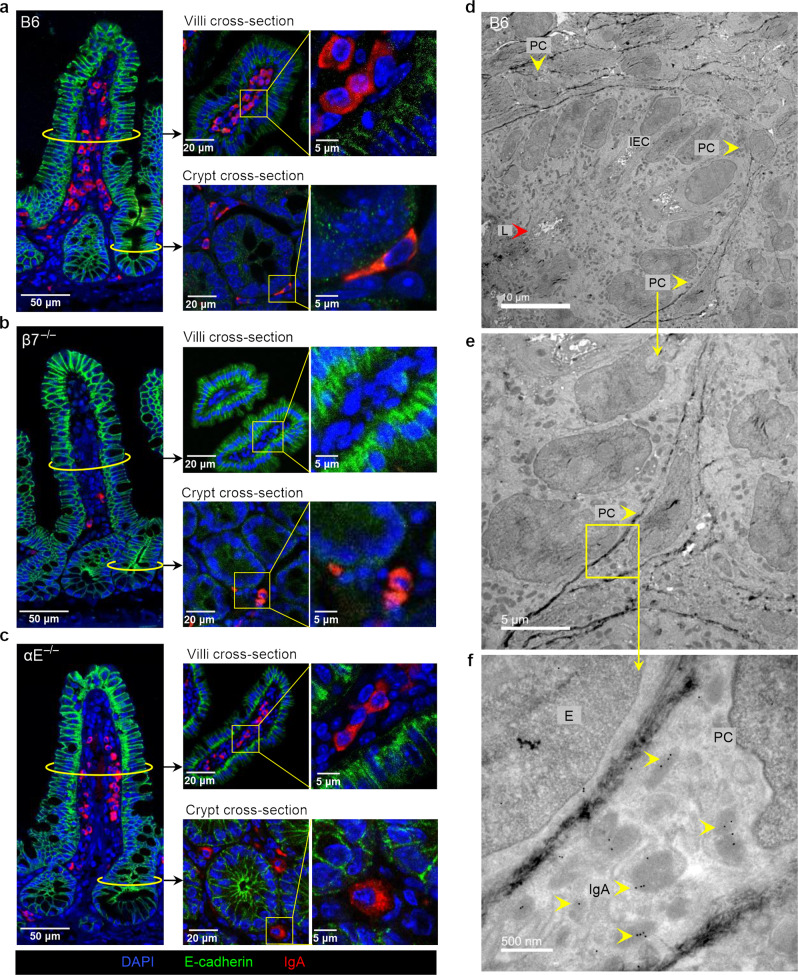
A subset of IgA + ASC acquire and elongated/sickled morphology near the crypt base of B6 mice but not in αE- or β7-deficient mice. **a** Round morphology of IgA + ASC in the mid-villous region, whereas a subset of IgA + ASC near the crypt base of B6 mice have an elongated (sickle-like) morphology (coronal and cross sections at indicated levels). **b** Absence of IgA + ASC in the mid-villous and near crypt base regions in β7^−/−^ mice. **c** Round morphology of IgA + ASC in the mid-villous region and around the crypt base of αE^−/−^ mice. Increasing magnification of the villous crypt base of B6 mice (**d**) Three cells (PC) with sickled morphology in contact with IEC (yellow arrowheads) (**e**) Higher magnification of an adherent cell with sickled morphology (**f**) Direct cell to cell contact between IEC and sickled cell with extensive RER and IgA immunogold particles (representative TEM images, L = lumen, E = epithelial cell).

Furthermore, in transversal cuts near the crypt base (crypt cross-section) of B6 mice we find IgA ASC that flatten against IEC and acquire a sickled appearance, as to increase their contact surface with IEC (Fig. 5a). The crypt base is identified by the small luminal diameter (Fig. 5d, L = lumen). Although the sickled appearance is obvious when present (Fig. 5a, crypt cross-section, Supplementary Fig. 4a, b) we defined these cells as >12 µm in length, to count them in an unbiased fashion (Supplementary Fig. 4b). Following these criteria, we did not find any elongated IgA + ASC in neither β7^−/−^ nor αE^−/−^ mice (Fig. 5b, c and Supplementary 4a, b, c). By contrast round non-adherent IgA + ASC (<12 µm length), were present in all strains. This supports the hypothesis that the ability of IgA + ASC to modify their morphology and flatten themselves in contact with IEC is dependent on αEβ7.

TEM, confirmed that cells with sickled morphology and extensive RER containing anti-IgA immunogold-bound particles abut basal crypt IEC (Fig. 5d–f). IgA and αE^+^ co-expression by dual immunogold particles confirmed that these were IgA + ASC in direct contact with IEC, co-expressing both αE and IgA (Supplementary Fig. 5). Thus, we demonstrate through three independent methods (TEM immunogold, flow cytometry and RNA-seq) that a subset of IgA^+^ ASC express integrin αEβ7 and establish direct contact with pIgR/E-cadherin-expressing IEC near the base of the ileal villus crypt.

## Discussion

The α4β7 integrin has emerged as a major therapeutic target for the inflammatory bowel diseases (IBD) and two drugs that target this integrin (i.e., natalizumab, vedolizumab) are used for the treatment of Crohn’s disease and ulcerative colitis.^34–36^ The specificities of these drugs are distinct, as natalizumab targets the shared α4 subunit on α4β7 and α4β1, while vedolizumab is specific for α4β7.^37^ Unexpectedly, phase III trials of a third drug (i.e. etrolizumab), that targets the shared β7 subunit on both α4β7 and αEβ7 did not show similar efficacy in patients with UC (https://www.roche.com/media/releases/med-cor-2020-08-10.htm.). Although the failure of a trial drug is multifactorial and cannot be easily attributed to any particular reason, it may illustrate the importance of understanding the potential cellular targets and biological implications of pathway blockade during physiology and pathology.

αEβ7 is expressed predominantly by T cells, a subset of dendritic cells within mucosal surfaces and by some upper respiratory tract B cells.^16^ However, despite αE/CD103 serving as a major diagnostic surface marker of malignant hairy cell leukemia B cells,^33^ the expression of αEβ7 by gut B cell lineage cells has not been reported to date.^18^ Herein, we describe an unreported population of IgA^+^ ASC that like IEL contact or intercalate within intestinal epithelial cells (IEC). Their elongated sickled morphology, particularly near the crypt base maximizes the surface area in direct contact with the basolateral side of the IEC. Direct relay of IgA to IEC for transcytosis near the crypt base could contribute to the protection of the stem cell niche from bacterial invasion. The ultrastructure of these ASC is consistent with that of a mature PC, as they have extensive rough endoplasmic reticulum (RER) containing IgA-bound immunogold particles. The potential relevance of the integrin for IgA transcytosis is highlighted by the fact that the luminal IgA deficit in αE-deficient mice is similar to that of β7-deficient mice and by the reduction in bacterial IgA coating. β7-deficient mice additionally have a marked B cell recruitment defect, likely due to their critical dependence on α4β7:MAdCAM-1 interactions for intestinal homing. Remarkably, they maintain luminal IgA levels comparable to αE^−/−^ mice. As part of potential compensatory mechanisms at play, we find higher IgA transcripts and increased pIgR expression in their terminal ilea. It is likely that other long-term adaptations such as differential PC maturation, fitness and/or longevity are participant as well.

Other explanations for a luminal IgA deficit, such as impaired IgA plasmablast recruitment, pIgR downregulation and a CD103^+^ DC/RA deficit, interfering with regulatory mechanisms or class switching were excluded by the recapitulation of the luminal IgA deficit in RAG^−/−^ mice transferred with CD103^−/−^ B cells. RAGs have intact RA production and a normal DC compartment.^21,22^ PIgR transcripts were clearly increased, suggesting that the epithelium senses the intraluminal IgA deficit, likely though TLR engagement of pathogen-associated molecular patterns, originating from an altered microbiota composition.^38^ In the ileal LP, we found 3 main B cell subsets, based on their expression of CD19 (shed upon class switching) and surface IgA. The CD19^+^ cells appeared to be in their earliest state of maturation as they had the highest nucleocytoplasmic ratio, expressed IgM, IgD, CCR7, and S1PR1. It is possible that these originate from ILF, as these cannot be removed as PP, prior to LPMC extraction. Those CD19^+^ that were class switched expressed AID and KI67, suggesting that they were still replicating. IgA + ASC which had shed C19 (CD19^neg^IgA^+^) (subsets 3 and 4) expressed transcripts consistent with terminal differentiation,^39^ such as Blimp-1, syndecan-1 (CD138), Xbp1 and BCMA, while subset 4 also expressed αEβ7.

Thus, we propose that as a late step in PC maturation, a subset of terminally-differentiated PC express αEβ7 to directly engage E-cadherin/pIgR-expressing epithelium in a manner reminiscent of IEL. Direct engagement of PC with IEC may represent an efficient mechanism for direct IgA relay, distinct from the canonical, yet still current model proposed by Brandtzaeg, in which ASC release IgA into the extracellular milieu to eventually reach pIgR through diffusion.^40^ The contribution of dIgA by αEβ7^+^PC to total luminal SIgA is difficult to quantify, as this subset may still be present in the LP of αE^−/−^ mice contributing to the total IgA that will passively reach PIgR for transcytosis. Of note, is that even in αE^−/−^ mice IgA ASC often line themselves along IEC within the villi, suggesting that they sense IEC-derived chemoatractants (e.g., CCL25) to approximate IEC. It would appear that proximity to IEC may optimize IgA transfer. The dynamics of the process is unknown, however we observe IgA ASC that appear to be at different stages of IEC engagement, suggesting that this might be a sequential process. Whether IEC engagement is final or these cells may disengage from one area and move to another remains to be ascertained. Given their surface receptor profile, it is likely that these cells are tissue residents and do not circulate.

**Fig. 6:**
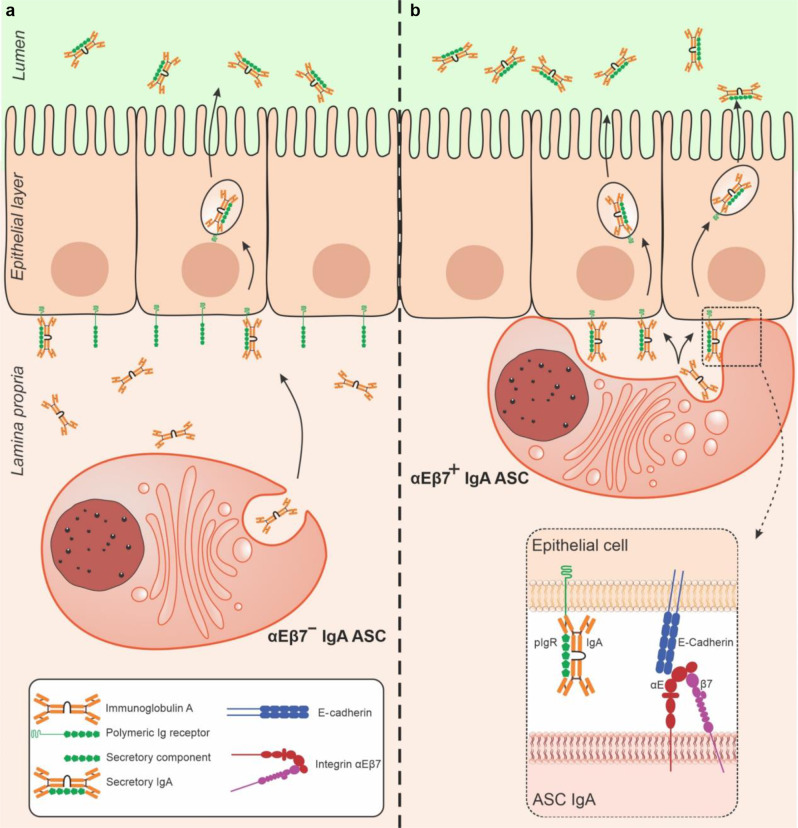
Alternate integrin αEβ7-independent and dependent models of IgA transcytosis. **a** Canonical diffusion model for IgA transcytosis in which dIgA is secreted by IgA + ASC into the extracellular milieu and diffuses to reach pIgR. **b** Proposed new model of αEβ7-dependent IgA transcytosis in which αEβ7 + IgA + ASC dock with IEC to directly relay dIgA to pIgR for transcytosis into the intestinal lumen.

We report on a subset of intestinal IgA + PC that express αEβ7, we demonstrate an unappreciated physiologic role of the integrin for the maintenance of luminal SIgA and propose an alternate mechanism for IgA transcytosis. In this αEβ7-dependent model, PC dock with intestinal IEC and directly transfer their IgA cargo to pIgR, for transfer to the intestinal lumen (Fig. 6). Whether interference with this unappreciated role for the integrin may be in part responsible for the lower efficacy of etrolizumab compared with the α4β7-specific agent (i.e., vedolizumab) in ulcerative colitis deserves further consideration.

## Materials and methods

### Mice

C57BL/6 (B6), C57BL/6-Itgb7tm1Cgn (Integrin β7^−/−^), B6.129S2(C)-Itgaetm1Cmp (Integrin αE^−/−^), B6.129S7-Rag1tm1Mom/J (Rag1^−/−^) mice were purchased from Jackson Laboratories (Bar Harbor, ME). PIgR^−/−^ mice were provided by Dr. Charlotte Kaetzel (University of Kentucky). IgA^−/−^ mice were provided by Dr. Lars Eckmann (San Diego Digestive Diseases Research Center). All mice were maintained under specific pathogen-free conditions and fed with a standard diet and water ad libitum during in vivo studies. Animal procedures were in accordance with governmental and institutional guidelines and approved by the Institutional Animal Care and Use Committees of the University of California San Diego and the San Diego VA Medical Center.

### Adoptive transfer studies

CD4^+^ T from the spleen and MLN of B6 mice and CD19^+^ B cells from B6, β7^−/−^ or αE^−/−^ mice were enriched by positive selection with anti-mouse CD4 microbeads (130-117-043, Miltenyi Biotec, Auburn, CA) or with anti-mouse CD19 microbeads (130-121-301, Miltenyi Biotec) respectively, as per the manufacturer’s instructions. B6 CD4 ^+ ^T cells (≥95% purity; 2.5 ×10^6^) were combined with CD19 ^+ ^B cells from B6, β7^−/−^ or αE^−/−^ mice (≥95% purity; 2.5 ×10^6^), suspended in 200 µL of PBS and injected intraperitoneally into 6-week-old RAG^−/−^ recipients. Tissues were harvested after 6 weeks once fecal IgA had reached B6 levels.

### Lymphocyte isolation

Splenocytes, mesenteric lymph node (MLN) lymphocytes were excised and rendered into a cell suspension by mechanical dissociation and sieving through wire mesh as previously described.^41^ Lamina propria (LP) mononuclear cells were isolated from terminal ileum.^42^ Briefly, tissues were flushed out fecal material with cold phosphate-buffered saline (PBS) (14190-144, Gibco, Carlsbad, CA), Peyer’s patches were removed, and collected in 30 ml of RPMI-1640 medium (21870-076, Gibco) supplemented with 10% heat-inactivated fetal calf serum (10082-147, Gibco), 10 mM glutamine,100 U/ml penicillin, 100 μg/ml streptomycin (10378016, Thermo Fisher Scientific, Waltham, MA). First, samples were washed three times with 1 mM EDTA (GR123, Hoefer, Holliston, MA) in HBSS (14170-112, Gibco) for 15 min at room temperature in a shaking incubator and once with HBSS without EDTA. Then, intestinal tissue was mechanical dissociated and enzymatically digested in 20 ml of RPMI-1640 medium containing 10% FBS, 1.5 mg/ml Collagenase A (C2139, Sigma-Aldrich, St. Louis, MO) and 0.5 mg/ml Dispase II (D4693, Sigma-Aldrich) for 30 min at 37˚C and 200 rpm in a shaking incubator. Following digestion, tissues were strained to yield a single-cell suspension, centrifuged 10 min at 1500 rpm at 4 °C. For in vitro stimulation assays, 2.5×10 ^5^ splenocytes were cultured in 96-well U-bottom plates with or without 50 µg/ml of LPS (00-4976-93, Invitrogen, Carlsbad, CA) for 72 h and stained for flow cytometry.

### Enzyme-linked immunosorbent assay (ELISA)

Fecal pellets were collected in 1 mL of phosphate buffer saline (14190-144, Gibco) and vortexed for 10 min. Particulate debris was removed after centrifugation at 4000 rpm and at 12,000 rpm for 10 min each. The supernatant was stored at −80 °C. Intestinal lamina propria IgA was obtained by fine mincing 1 cm of ileum in PBS with protease Inhibitor (S8820-20TAB, Sigma, Life Science) followed by homogenization and storage at −80 °C. For normalization, total protein concentration of the soluble fractions was measured by a Coomassie (Bradford) Protein Assay Kit (23200, Thermo Fisher Scientific). IgA levels were measured using a commercial ELISA Kit (88-50450-86, Thermo Fisher Scientific) as per manufacturer’s instructions. Analyte concentrations were determined by comparison with standards, by using a standard curve generated as a 4-parameter curve fit to determine ELISA kit assay values.

### Flow cytometry

Cells from indicated compartments were suspended in PBS with 1% fetal bovine serum, pre-incubated with anti-mouse CD16/32 (Fc-block, Clone 93, eBiosciences, San Diego, CA) and stained with anti-mouse fluorochrome-conjugated antibodies (listed in Table S1). UltraComp eBeads™ Compensation Beads (01-2222-42, Invitrogen) were used for compensation. Acquisition of samples were performed using a Cytek Northern Light cytometer (Cytek, Fremont, CA). Flow-cytometry data were analyzed using the FLOWJO software (Tree Star, Ashland, OR).

### Analysis of fecal IgA bacterial coating

Fecal pellets were collected in 1 mL of phosphate buffer saline (14190-144, Gibco), vortexed for 10 min, homogenized, and centrifuged at 400 × *g* to remove large debris. Supernatant was filtered through a sterile 70 μm strainer and centrifuged at 8000 × *g*. The bacterial pellet was resuspended in PBS 0.25% BSA with SYTO BC (Thermofisher) and incubated for 30 min on ice. Then, bacteria were stained with PE anti-mouse IgA (clone: mA-6E1, 10 μg/ml) for 20 min on ice, washed and resuspended in PBS 0.25% BSA with DAPI (Life Technologies) prior to flow cytometry using a low FSC and SSC threshold to allow bacterial detection.

### RNA extraction, cDNA synthesis, and real-time PCR

Total RNA was isolated using RNeasy® Plus Mini kit (QIAGEN, Hilden, Germany) and cDNA was synthesized from 2000 ng of total RNA using High Capacity cDNA Reverse Transcription Kit (Applied Biosystems, Foster City, CA) on a PTC-200 Thermal Cycler (Marshall Scientific, Hampton, NH). Pigr gene expression (Assay ID Mm00465049_m1, Thermo Fisher Scientific) was performed using TaqMan® Fast Universal PCR Master Mix (Applied Biosystems) and Gapdh gene expression (Assay ID Mm99999915_g1, Thermo Fisher Scientific) was amplified as endogenous controls using Step One Plus Instrument (4376600, Applied Biosystems). Relative Pigr gene expression was calculated from reference gene using CT values obtained from Applied BioSystems’ StepOne Software (Version v2.3).

The mRNA expression levels of IgA was performed using SYBR®FAST qPCR kit (cat. #4385612, Applied Biosystems, CA) with thermal conditions of 20-s preincubation at 95 °C followed by 40 cycles at 95 °C for 3 s and 60 °C for 30 s. Gene expression was calculated using from reference gene using CT values obtained from Applied Biosystems’ StepOne Software (Version v2.3) and using GAPDH as reference gene. The following primers were used to quantify transcripts in the tissue samples: immunoglobulin A (IgA) F: 5ʹCCTAGTGTTTGAGCCCCTAA3ʹ and IgA R: 5ʹGGAAGTGCAGGGATACTTTG3ʹ; (GAPDH) F: 5ʹGATTCCACCCATGGCAAATTC3ʹ and GAPDH_R: 5ʹTGGGATTTCCATTGATGACAAG3ʹ.

### Immunostaining and confocal laser scanning microscopy acquisition

Distal ileum (10 cm) samples were opened, fixed in 10% neutral buffered formalin, embedded in paraffin on edge, and cut into 4-μm-thick sections. For immunofluorescence microscopy, sections were deparaffinized, rehydrated, and placed in a pressure cooker set to high pressure and heated for 20 min with Tris-EDTA pH 9.0 antigen retrieval buffer. The slides were stained using a 3D-printed Freequenza Rack (https://3dprint.nih.gov/discover/3dpx-012172) and Shandon Plastic Coverplates (72110017, Thermo Fisher Scientific). After antigen retrieval slides were blocked in 5% normal donkey serum, 0.3% Triton X-100 in PBS for 1 h at RT, and incubated with primary pIgR antibody in PBS-T (0.1% Tween 20) for 18 h at 4 °C, washed in PBS-T, and stained with an anti-goat secondary for 1 h at RT. The slides were then washed and incubated for another 18 h at 4 °C with either IgA and CD31 or IgA and E-cadherin, washed with PBS-T, and stained with secondaries for 1 h at RT. After another wash, they were counterstained with Hoechst (H3570, Thermo Fisher Scientific) 1:1000 in PBS. The slides were removed from the Freequenza rack and treated with TrueBlack Lipofuscin Autofluorescence Quencher (23007, Biotium, Fremont, CA) 1:20 in 70% reagent alcohol, for 30 sec. The quencher was removed, and slides were washed in PBS. Specimens were mounted with ProLong Gold Antifade Mountant (P36930, ThermoFisher Scientific) and stored at RT. All antibodies details are listed in Table S2. Image acquisition was performed with a ZEISS LSM780 confocal microscope (Thornwood, USA) equipped with a 40x/1.3 NA EC Plan-Neofluor oil objective. High-resolution images were taken through a tile scan. Each square region was 610 µm per side with a pixel size of 0.13 µm.

### TEM immunogold labeling

For immunoelectron microscopic studies, distal ilea were fixed for 12 h in 4% PFA in 0.1 M phosphate buffer, pelleted in 10% gelatin and cryoprotected by infusion with 2.3 M sucrose overnight at 4 °C. One mm^3^ tissue blocks were mounted onto specimen holders and snap frozen in liquid nitrogen. Ultrathin cryosections (70–80 nm) were cut, placed on a 1:1 mixture of 2.3 M sucrose and 2% methyl cellulose (15cp) and transferred onto Formvar and carbon-coated copper grids.

Tissue grids were placed on 2% gelatin at 37 °C for 20 min, rinsed with 0.15 M glycine/PBS and the sections were blocked using 1% cold water fish-skin gelatin. Ultrathin sections were incubated for 2 h at RT with purified rat anti-mouse IgA antibody (Clone C10-3. BD Pharmingen) diluted at 1:500 and rabbit anti-mouse E-Cadherin antibody (Clone 24E10, Cell Signaling), washed and followed by an 1 h incubation with 12 nM gold conjugated goat anti-rat IgG (Jackson ImmunoResearch Inc.) and 16 nm gold conjugated goat anti-rabbit IgG (Jackson ImmunoResearch Inc., Westgrove, PA), diluted 1:20 in 1% BSA/PBS. Sections were post-fixed for 5 min with 1% glutaraldehyde in PBS, washed thoroughly with distilled water, and then contrasted (10 min in 0.4% uranyl acetate and 1.8% methyl cellulose on ice). Grids were viewed using a JEOL 1400plus (JEOL, Peabody, MA) transmission electron microscope and photographed using a Oneview 4KGatan digital camera (Gatan, Pleasanton, CA).

### Quantification of pIgR immunofluorescent signal

Images were processed with ImageJ (Fiji) software.^43^ To quantify PIgR expression, values were obtained individually for the villus-crypt axis using a region of interest (ROI). First, free-hand ROI were created by delimiting the axis according to DAPI and E-cadherin label. This step was done by a masked observer (ZM) who was blinded to PIgR IF signal. Then, a macro was used to apply the ROI to the PIgR fluorescent image, the product of the area and mean intensity (Int Den) was recorded and expressed per each high-resolution image. At least 5 villus-crypt axis per image were analyzed.

### IgA ASC morphology assessment

The length of IgA + ASC was assessed following a semi-automatic method using ImageJ software. Briefly, a binary image was created applying a uniform IgA IF signal threshold to all images, which allowed generation of an ROI for each IgA cell. Then, a straight line was manually drawn along each ROI according to the maximum length of each cell, which automatically provides length in microns. Thirty or more cells were measured near the pericryptal base region for each mouse strain. The crypt base was identified by its small luminal space.

### Cell sorting

Single-cells suspension of LPMC were depleted of CD3^+^ cells using CD3ε MicroBead Kit (130-094-973, Miltenyi Biotec) and LS columns (130-042-401, Miltenyi Biotec) and stained with anti-mouse CD19 PerCpCy5.5 (115534, Biolegend, San Diego, CA), anti-mouse CD3 APC-eFluor 780 (47-0032-82, Invitrogen), anti-mouse IgA PE (12-4204-83, Invitrogen), anti-mouse CD103 APC (17-1031-82, Invitrogen) and anti-mouse Integrin B7 BV421 (564283, BD Bioscience, La Jolla, CA) and sorted on a fluorescence‐activated cell sorter FACSAria (BD Biosciences) using FACSDiva software (BD Biosciences).

### Transmission electron microscopy and nucleo-cytoplasmic ratio analysis

Cells were fixed with 2% glut. in 0.10 M cacodylate buffer and further postfixed in 1% OsO4 in 0.1 M cacodylate buffer for 1 h on ice. Cells were stained with 2% uranyl acetate for 1 h on ice, dehydrated in ethanol (50–100%) on ice followed by 2 washes with acetone (10 min each) and embedded with Durcupan (44611, Sigma Aldrich). Sections were cut at 60 nm on a Leica UCT ultramicrotome (Wetzlar, Germany), and placed on 300 mesh copper grids. Sections were post-stained with 2% uranyl acetate for 5 min and Sato’s lead stain for 1 min. Images were obtained by using Jeol 1400 plus Transmission Electron Microscope equipped with Gatan digital camera (Peabody, MA). The nucleus/cytoplasm ratio analysis was performed with ImageJ software (Fiji). The calculation was then carried out by applying two regions of interest (ROIs), one delimiting the total area per cell and other delimiting the nuclear area corresponding to each one.

### Bulk RNA-sequencing and analysis

Total cellular RNA was extracted from the 4 sorted populations: CD19^+^ IgA^−^, CD19^+^ IgA^+^, CD19^−^IgA^+^CD103^−^ and CD19^−^IgA^+^CD103^+^ in TRIzol reagent (15596018, Invitrogen). RNA library preparation was conducted using a TruSeq RNA Library Prep Kit v2 for Illumina (RS-122-2001, Illumina, San Diego, CA) according to the manufacturer’s protocols. After quality assessment, sequencing was carried out on a NovaSeq 6000 (Illumina) using single-ended 50-bp reads. Quality control of the raw fastq files was performed using the software tool FastQC v0.11.3. Sequencing reads were trimmed with Trimmomatic v0.36 and aligned with the mouse genome (GRCm38) using the STAR aligner v2.5.3a. Read quantification was performed with RSEM^44^ v1.3.0 and the M19 Gencode annotation.^45^ The R BioConductor packages edgeR and limma were used to implement the limma-voom method for differential expression analysis. In brief, lowly expressed genes—those not having counts per million (cpm) ≥3 in at least 3 of the samples—were filtered out and then trimmed mean of M-values (TMM) normalization was applied. The experimental design was modeled upon condition and batch (~0 + condition + batch). The voom method was employed to model the mean-variance relationship in the log-cpm values weighted for inter-subject correlations in repeated measures of mice, after which lmFit was used to fit per-gene linear models^46^. Empirical Bayes moderation was applied with the eBayes function. Significance was defined by using an adjusted *p* value cut-off of 0.05 after multiple testing correction using a moderated t-statistic in limma. Functional enrichment of the differentially expressed genes was performed using WebGestalt^47^ (including GSEA^48^), GSVA^49^ and SPIA.^50^

### Statistical analysis

Results are expressed as mean ± S.D. unless otherwise indicated. One- or two-way analysis of variance with Bonferroni or Dunnett´s post-hoc tests were used to compare groups. Significance was set at *p* < 0.05 and two-tailed tests were used in all experiments. Calculations were performed using GraphPad Prism version 8 software (GraphPad Software, La Jolla, CA).

## Supplementary information

Supplementary figures
