# Supplementary material for: An integrin αEβ7-dependent mechanism of IgA transcytosis requires direct plasma cell contact with intestinal epithelium
Source: Mucosal Immunol. 2021 Aug 20;14(6):1347–57. doi: 10.1038/s41385-021-00439-x (PMC8528714; doi:10.1038/s41385-021-00439-x)
Supplement: Supplementary file 1 — Supplementary figures [file 41385_2021_439_MOESM1_ESM.pdf]

An integrin αEβ7-dependent mechanism of IgA transcytosis requires plasma cell contact with intestinal epithelium

Mauricio Guzman, Luke R. Lundborg, Shaila Yeasmin, Christopher J. Tyler, Nadia R. Zgajnar, Vanessa Taupin, Katarzyna Dobaczewska, Zbigniew Mikulski, Giorgos Bamias and Jesús Rivera-Nieves*

Supplementary Information:

Supplementary Figures 1-6

Tables 1-3

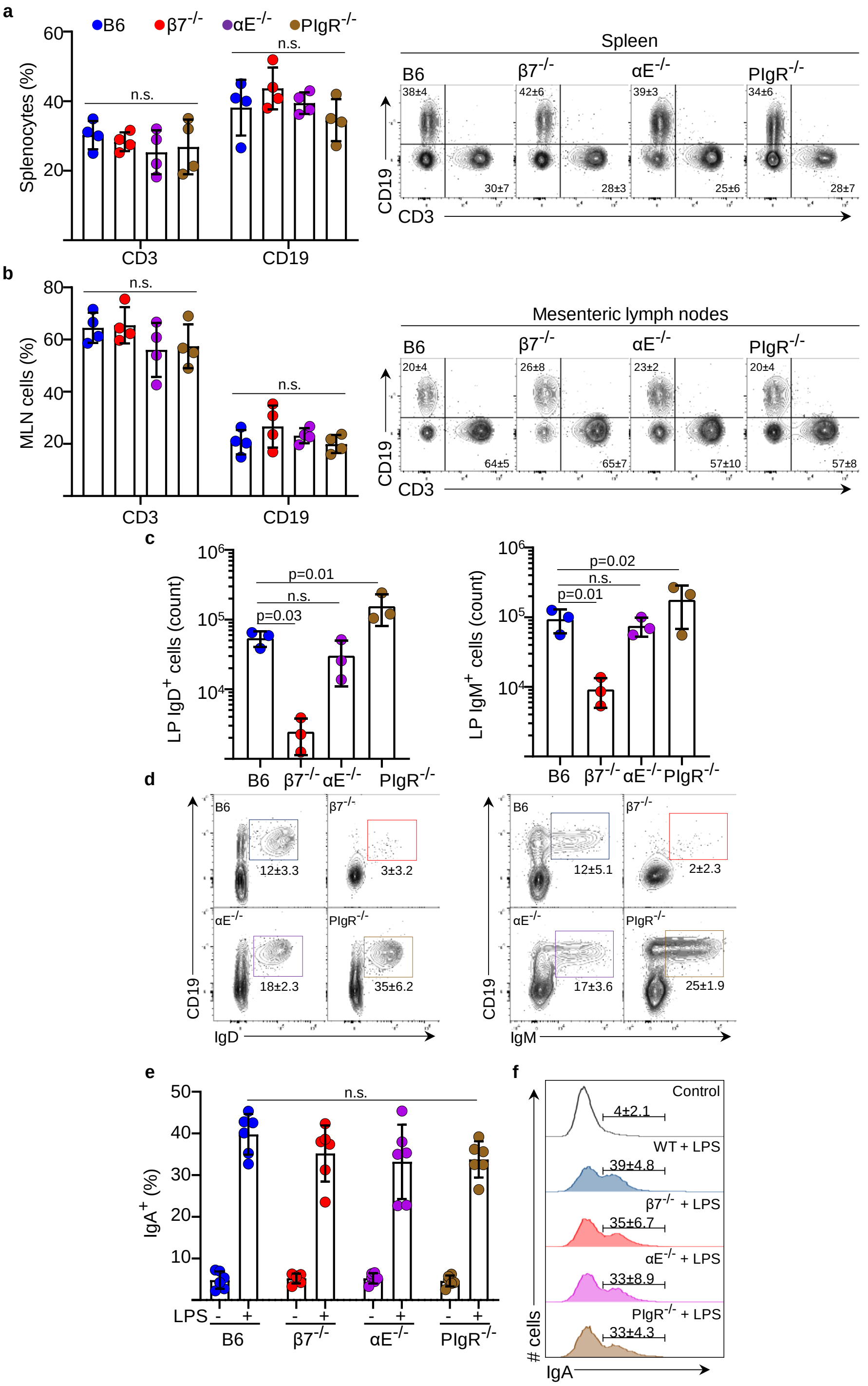

Figure S1. **T and B cell recruitment to spleen and MLN are normal in all mouse strains whereas intestinal IgD+ and IgM+ ASC recruitment is compromised only in β7$^{-/-}$ mice. (a, b)** Percentages and representative plots of CD3$^+$ and CD19$^+$ cells in the spleen and MLN (n= 4/strain, statistical significance determined using ANOVA, followed by Tukey's multiple comparison test). **(c, d)** Absolute counts of IgD$^+$ and IgM$^+$ within the ileal LP and representative contour plots (n=3/strain, statistical significance determined using ANOVA, followed by Tukey's multiple comparison test). **(e)** Percentage of IgA$^+$ cells after *in vitro* stimulation of splenocytes with or without LPS for 72 h **(f)** representative histogram plots (mean ± SD, n=3-6 mice per group from three independent experiments).

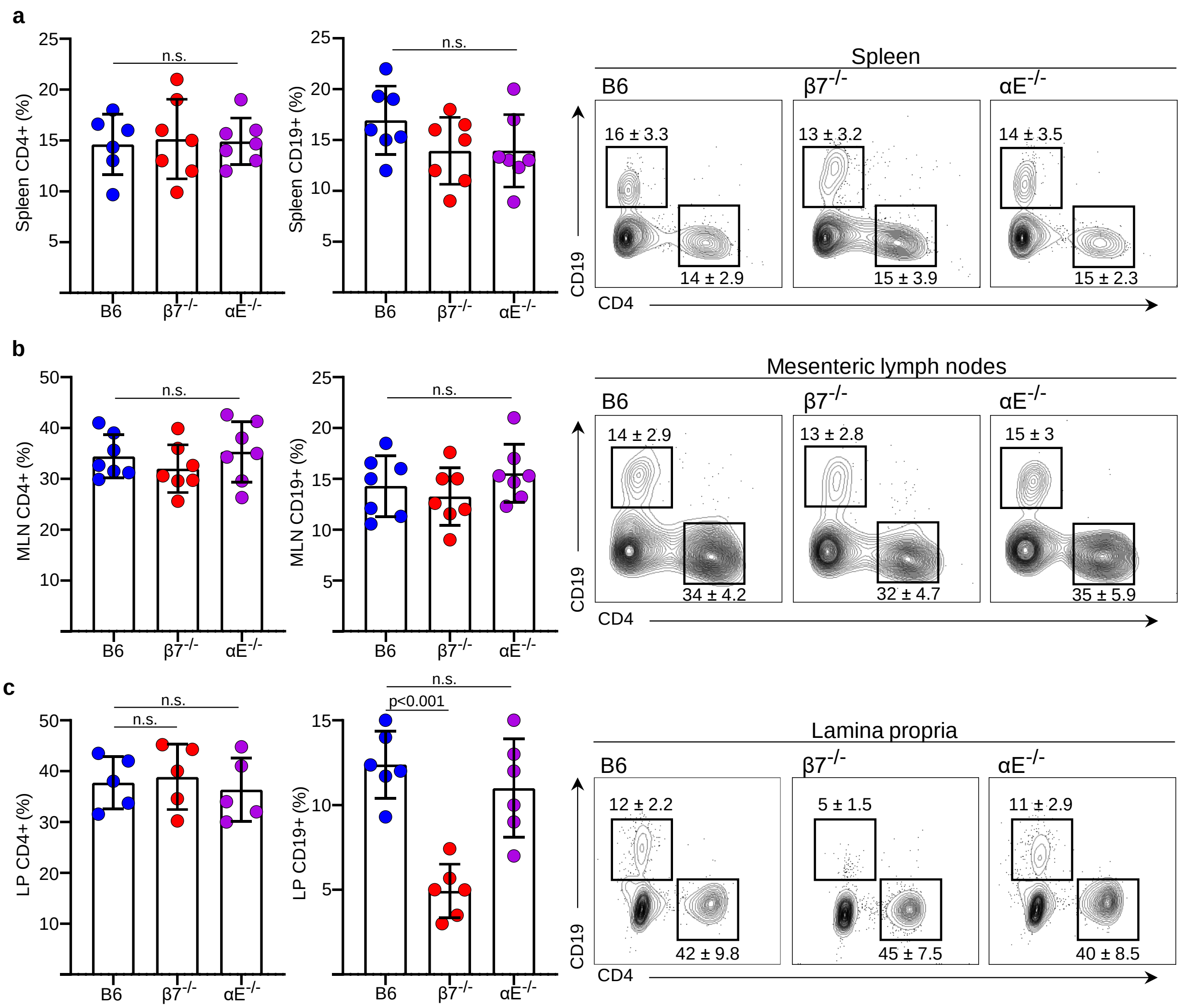

Figure S2. T and B cell reconstitution of spleen, MLN and LP is normal in RAG1$^{-/-}$ mice receiving CD4^{+} T cells from all strains, while only β7$^{-/-}$ B cells are unable to reconstitute the LP compartment. (a, b, c) Percentages and representative contour plots of CD4^{+} and CD19^{+} cells within **(a)** spleen, **(b)** mesenteric lymph nodes and **(c)** ileal lamina propria of reconstituted RAG$^{-/-}$ mice. (mean ± SD, n=5-7 mice, statistical significance determined using ANOVA, followed by Tukey's multiple comparison test).

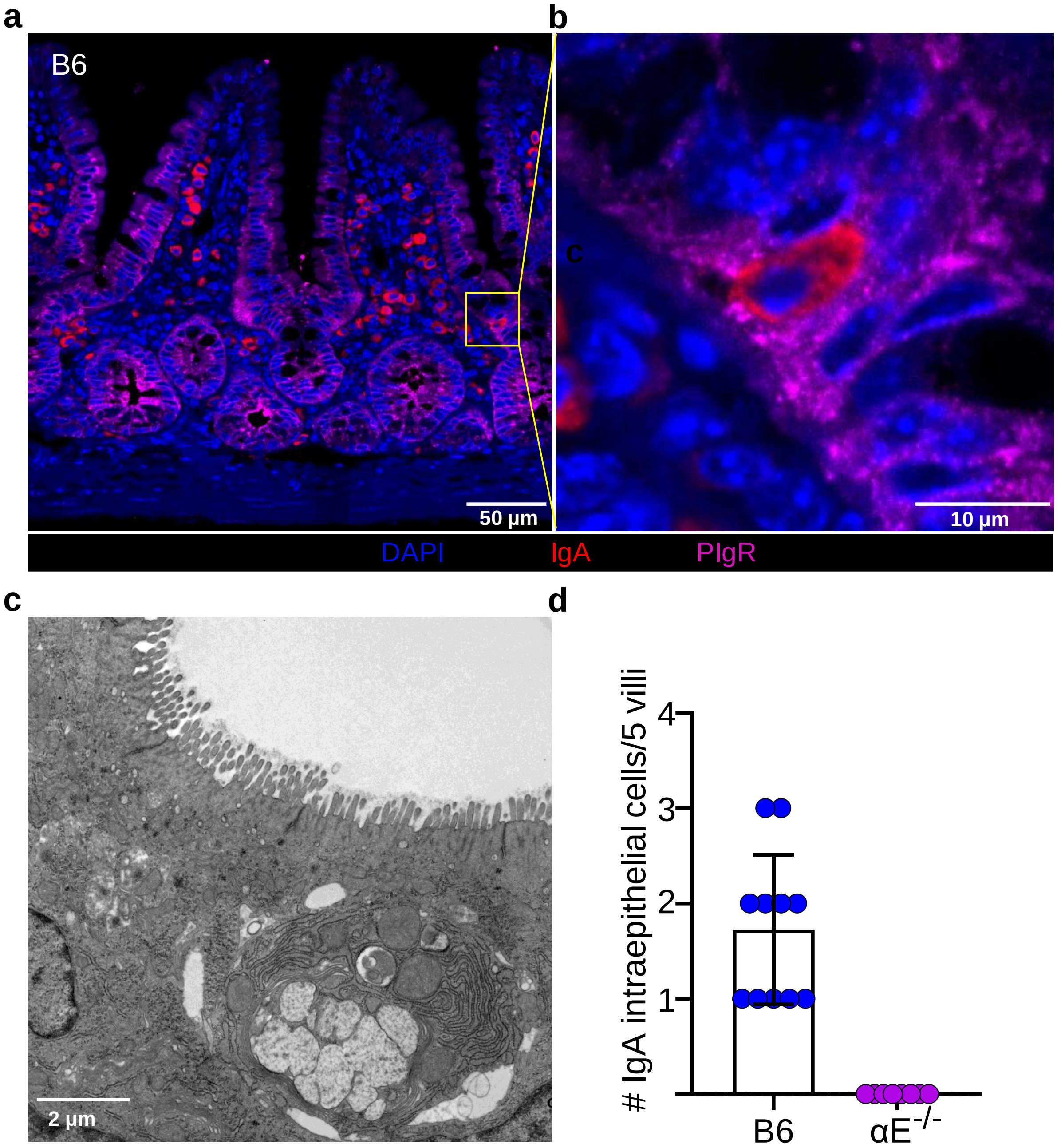

Figure S3. Intraepithelial IgA ASC interdigitate within pIgR-expressing IEC near the crypt base of B6 mice yet are absent in αE-/- mice. (a) IF staining of IgA+ ASC and pIgR (representative images of terminal ileum) **(b)** High magnification IF image and **(c)** TEM image of IgA+ ASC infiltrating epithelium. **(d)** Number of intraepithelial IgA+ ASC per 5 terminal ileal villi.

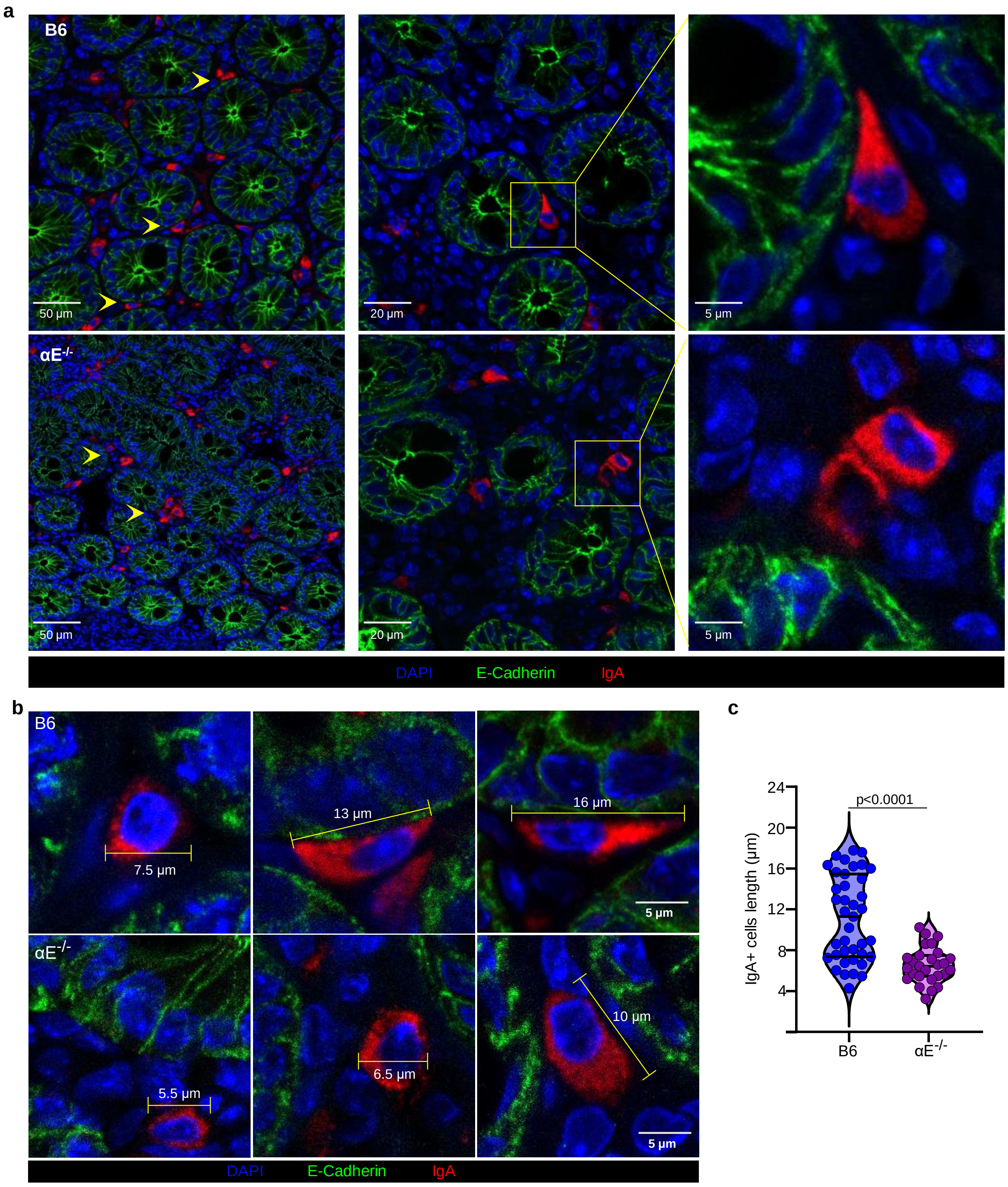

Figure S4. IgA+ ASC acquire a sickled morphology in the pericryptal region of B6 mice but not in CD103-/- mice. (a) Representative IF images of IgA+ ASC and E-cadherin show sickled/flattened morphology and IEC adherence in B6 mice. (**b**) Representative images of the morphology and length assessment of IgA+ ASC in indicated strains. **(c)** IgA+ ASC > 12 μm are absent in ileal LP of αE-/- mice. Violin plot of median and quartiles, n> 30 cells from 3 independent experiments, p calculated by Student's t-test.

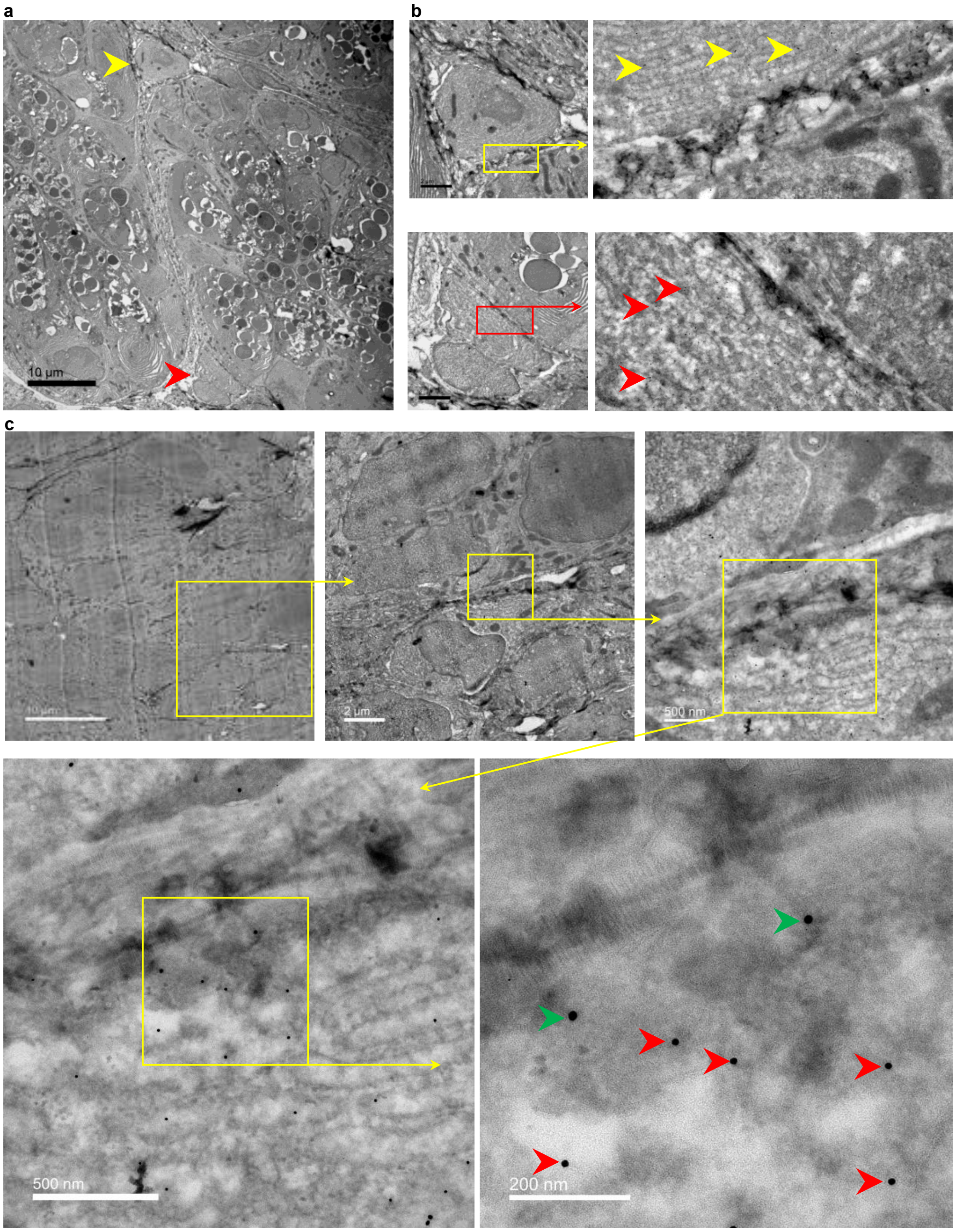

Figure S5. A subset of adherent IgA+ ASC express integrin αE and establish direct contact with intestinal epithelial cells. (a) TEM images of cross sections through the crypt base show 2 adherent cells (arrowheads) in contact with epithelial cells (likely Paneth cells) **(b)** Higher magnification shows cell to cell contact and immunogold particles within RER of adherent cells. **(c)** TEM images demonstrate co-expression of IgA (red arrows, 12nm immunogold particles) and integrin αE (green arrows, 18nm immunogold particles) in adherent cells. Increasing magnification of the yellow-box area. (Representative images of cross sections through the ileal pericryptal base from three B6 mice).

Table S1: Antibodies used for flow cytometry.

Target	Clone	Conjugate	Vendor	Product number
CD3	17A2	APC-eFluor 780	eBioscience	47-0032-82
CD19	eBio1D3	PerCO-Cy5.5	eBioscience	45-0193-82
IgA	mA-6E1	PE	eBioscience	12-4204-82
IgD	11.26c.2a	BV650	Biolegend	405721
IgM	RMM-1	BV605	Biolegend	406523
IgG	Poly	BV510	Biolegend	405331
CD49d	R1-2	BV480	BD Bioscience	746526
Integrin β7	FIB504	BV421	BD Bioscience	564283
CD103	2E7	AlexaFluor 647	Biolegend	121410
Live dead		Zombie NIR dye	Biolegend	423106

Table S2: Antibodies used for immunostaining and confocal laser scanning microscopy.

Target	Host	Clone	Vendor	Reference	Concentration
Mouse IgA	Rat	C10-3	BD Pharmingen (La Jolla, CA)	556969	2.5 ug/ml
Mouse E-Cadherin	Rabbit	24E10	Cell Signaling Technology (Danvers, MA)	3195s	2 ug/ml
Mouse pIgR	Goat	Polyclonal	R&D System (Minneapolis, MN)	AF2800	2 ug/ml
Mouse CD31	Armenian hamster	2H8	Millipore Sigma	MAB1998 Z	2 ug/ml
Alexa Fluor 488 anti-rabbit (H+L)	Goat	Polyclonal	Jackson ImmunoResearch	111-545-144	2 ug/ml
Alexa Fluor 488 anti-Armenian hamster IgG (H+L)	Goat	Polyclonal	Jackson ImmunoResearch	127-545-160	2 ug/ml
Alexa Fluor 594 anti-rat IgG (H+L)	Goat	Polyclonal	Invitrogen	A-11007	2 ug/ml
Alexa Fluor 647 anti-goat	Donkey	Polyclonal	Invitrogen	A-21447	2 ug/ml

Table S3: Antibodies used for immunoelectron microscopy.

Target	Host	Clone	Vendor	Reference	Concentration
Mouse IgA	Rat	C10-3	BD Pharmingen (La Jolla, CA)	556969	2.5 ug/ml
Mouse CD103	Rabbit	Polyclonal	Invitrogen	PA5-99400	2 ug/ml
12 nm Colloidal Gold AffiniPure Goat Anti-Rat IgG	Goat	Polyclonal	Jackson ImmunoResearch	112-205-143	1:20
18 nm Colloidal Gold AffiniPure Goat Anti-Rabbit IgG	Goat	Polyclonal	Jackson ImmunoResearch	111-215-144	1:20
